# Effect of Patient-Physician Relationship on Withholding Information Behavior: Analysis of Health Information National Trends Survey (2011-2018) Data

**DOI:** 10.2196/16713

**Published:** 2020-01-29

**Authors:** Xin Yang, Jason Parton, Dwight Lewis, Ning Yang, Matthew Hudnall

**Affiliations:** 1 Institute of Business Analytics The University of Alabama Tuscaloosa, AL United States; 2 Department of Information Systems, Statistics, and Management Science The University of Alabama Tuscaloosa, AL United States; 3 Department of Management The University of Alabama Tuscaloosa, AL United States

**Keywords:** withholding information behavior, patient-physician relationship, electronic medical records, privacy

## Abstract

**Background:**

Patients’ withholding information from doctors can undermine medical treatment, create barriers for appropriate diagnoses, and increase systemic cost in health care systems. To date, there is limited literature detailing the association between trends of patients withholding information behavior (WIB) and the patient-physician relationship (PPR).

**Objective:**

The aim of this study was to explore the prevalence trend of WIB after 2011 and examine the effects of PPR on WIB and its time trend.

**Methods:**

A total of 5 iterations of data from the Health Information National Trends Survey (years: 2011-2018; n=11,954) were used to explore curvilinear trends of WIB among the US population. Multiple logistic regression models were used to examine curvilinear time trends of WIB, effects of PPR on WIB, and moderation effects of PPR on the WIB time trend.

**Results:**

The WIB prevalence has an increasing trend before 2014, which has the highest rate of 13.57%, and then it decreases after 2014 to 8.65%. The trend of WIB is curvilinear as the quadratic term in logistic regression model was statistically significant (*P*=.04; beta=−.022; SE=0.011; odds ratio [OR] 0.978, 95% CI 0.957-0.999). PPR is reversely associated with WIB (*P*<.001; beta=−.462; SE=0.097; OR 0.630, 95% CI 0.518-0.766) and has a significant moderation effect on time trends (*P*=.02; beta=−.06; SE=0.025; OR 0.941, 95% CI 0.896-0.989). In general, poor quality of PPR not only significantly increased the WIB probability but also postponed the change of point for WIB curvilinear trend.

**Conclusions:**

Findings suggest that the time trend of WIB between 2011 and 2018 is curvilinear and moderated by the quality of the PPR. Given these results, providers may reduce WIB by improving PPR. More research is needed to confirm these findings.

## Introduction

### Background

Medical literature recognizes that quality communicative interactions between patients and their doctors are important to the success of medical treatment [1]. Many medical conditions can be accurately diagnosed by providers in an efficient manner if patients’ medical history is fully divulged [2] and clinicians express some empathy during medical visits [3]. As such, limited disclosure of critical health information between patients and doctors serves as a barrier to the success of a prescribed medical regimen. Despite this intuitive knowledge, existing evidence suggests that patients purposefully withhold relevant medical information from clinicians [4].

The reasons for patients withholding information are multifold. Several studies have examined factors that influence withholding information behavior (WIB), including demographics [5], social economics [6], and health [7]. Additional reasons include embarrassment of maladaptive health behavior and aversion to sensitive medical topics that patients are reluctant to share comprehensive details about their health status [4]. Recent medical data breaches are another possible reason why patients may not share all information and materials related to their medical history. In particular, prior studies highlight that individuals are more likely to withhold information when there are increased concerns about security [8]. Between the years 2009 and 2018, there were 2546 reported health care data breaches, which affected the health care records of 189,945,874 patients [9]. Given the increase in prevalence of medical data breaches across the United States as well as the potential influence of patients’ perceptions on medical data security via a willingness to share their medical history, an understanding of patients’ withholding patterns is warranted. To date, the scholarly findings are limited on the topic of patients’ WIB and its prevalence time trend in recent years. Evidence by Patel et al suggests that the proportion of US patients who exhibited medical WIB were 7%, 8%, and 5% for the years 2012, 2013, and 2014, respectively, indicating that the time trend (ie, slope) of WIB prevalence may not be linear or moving in a specific direction [10]. However, Patel et al’s [10] findings appear limited because of the study’s relatively small sample size and period, and the United States has witnessed a fair number of health care data breaches since publication. From our searches, we have found no additional studies that either support or conflict with these findings. Updated findings examining WIB that include more periods may provide better insights for policy-making state and national health officials.

Up to now, there is a paucity of literature on how patient-physician relationships (PPRs) influence patients’ WIB. PPR, which is measured by time spent, understanding, involvement, and helpfulness [11,12], is thought to be positively related with the intention to share protected health information (PHI) [7]. In a qualitative study among a small group of female Latina patients, 26 out of 28 study participants reported that their willingness to disclose health information depends on a PPR including spending enough time with patients, involving patients in decisions, and paying attention to patients’ feelings and emotions [13]. In a phenomenology (ie, the sciences of how people experience phenomena) study, factors such as being involved, listening attentively, and leaving time for patients influenced participants to disclose their use of traditional and complementary medicine to their doctors [14]. However, to the best of our knowledge, no study has empirically examined how PPRs influence patients’ withholding behavior and its longitudinal trend in a nationwide representative sample of the US population. An examination of the effects of PPR on WIB time trends may provide valuable insights for policy makers as well as providers to inform them on potential needs to adjust communicative practices with prospective patients.

### Objectives

Given the previously mentioned knowledge gaps about WIB in the United States, we investigate the longitudinal prevalence of WIB and the moderating effects of PPR on WIB trends adjusting for variables well studied in previous research. Using 2011 to 2018 responses from the Health Information National Trends Survey (HINTS), we sought to investigate the following areas:

Q1: Is there a stable pattern of WIB among patients over time?

Q2: Does increased self-reported satisfaction of a patient’s relationship with their provider meaningfully lower the odds that a patient would withhold important medical information during treatment?

Q3: Does the PPR influence the national trajectory of WIB over time?

We believe that findings from this study could contribute to shifting the paradigm in medical counseling research in several aspects. First, technological advancements and setbacks in the medical field have a dynamic and arguably instantaneous relationship with population health behavior [15]. Therefore, studies examining WIB and associated factors may be more informative if investigated over time. Moreover, increased knowledge of the influence that the PPR has on WIB may help health officials to allocate resources for behavioral and counseling-related patient-provider interventions to promote population health outcomes in the United States.

## Methods

### Sample

Findings from this study were derived using the following 5 waves of responses from HINTS: 2011(HINTS 4 cycle 1), 2012 (HINTS 4 cycle 2), 2014 (HINTS 4 cycle 4), 2017 (HINTS 5 cycle 1), and 2018 (HINTS 5 cycle 2). HINTS is an ongoing serial cross-sectional survey conducted by the National Cancer Institute to document attitudes and perceptions about health information access and use among noninstitutionalized US adults. The primary inclusion criteria for HINTS waves were based on the availability of survey questions focused on WIB. Participants self-reporting no visits to a nonemergency provider during the past 12 months were also excluded from analyses as these individuals would not have the opportunity to demonstrate WIB. A total sample of 11,954 respondents with complete records across 5 survey years, which represents about half US population each year, was used for descriptive analyses and logistic regression models.

### Dependent Variable

Our outcome of interest (WIB) was obtained from the HINTS survey item: “Have you ever kept information from your health care provider because you were concerned about the privacy or security of your medical records? (Yes/No)” (Question D3, HINTS 5 cycle 2).

### Patient-Physician Relationship

The PPR was operationalized using responses from the following 7 items in our study: (1) “How often did they give you the chance to ask all the health-related questions you had?,” (2) “How often did they give the attention you needed to your feelings and emotions?,” (3) “How often did they involve you in decisions about your health care as much as you wanted?,” (4) “How often did they make sure you understood the things you needed to do to take care of your health?,” (5) “How often did they explain things in a way you could understand?,” (6) “How often did they spend enough time with you?,” and (7) “How often did they help you deal with feelings of uncertainty about your health or health care?.” Participant’s responses to PPR questions were measured using a Likert-type scale scored as follows: 1=always, 2=usually, 3=sometimes, and 4=never. In a previous study, these 7 items were used to define PPRs with a Cronbach alpha of .94 and composite reliability of 0.9 in confirmatory factor analysis [8]. In order to improve interpretability, we first reverse coded the 7 items (1=never, 2=sometimes, 3=usually, 4=always). To make logistic regression intercept meaningful, we substracted the reverse coded 7 items by 1 so that these 7 items had the minmum score of 0 (0=never, 1=sometimes, 2=usually, 3=always). A factor-based score reflecting PPR was generated by the mean of 7 recoded items’ scores and used as an independent variable in the logistic regression models [16]. To help readers internalize the context of our findings, the PPR was ordinally categorized into tertiles as “poor” (tertile 1: 0-2.14), “fair” (tertile 2: 2.15-2.85), and “good” (tertile 3: 2.86-3) in descriptive analyses. The PPR remained as a continuous variable in models adjusted for key covariates to optimize the statistical power in the study’s findings.

### Other Variables

The time when surveys were conducted (2011, 2012, 2014, 2017, and 2018) was used to investigate the time trend aspect. Demographic variables include gender (male/female), age group (18-24, 25-44, 45-64, and 65+ years), census division (New England, Middle Atlantic, East North Central, West North Central, South Atlantic, East South Central, West South Central, Mountain, and Pacific), born in the United States (yes/no), urbanity (urban and rural), occupation (employed, unemployed, retired, disabled, and other), race or ethnicity (Hispanic, non-Hispanic white, non-Hispanic black, non-Hispanic other, and non-Hispanic Asian), and education (less than high school, 12 years or completed high school, some college, and college graduate or higher). Participants’ health status items include self-reported general health (excellent, very good, good, fair, and poor), ever had cancer (have you ever been diagnosed as having cancer?: yes/no), and depression and anxiety index calculated and categorized using Patient Health Questionnaire-4 [17]. A perceived provider using electronic health (eHealth) records system was assessed as “Do any of your doctors/HCP maintain your medical records in a computerized system? (yes/no).” Trust of a doctor is evaluated by the item “In general, how much would you trust information from a doctor?” (a lot, some, a little, and not at all). The frequency of visiting a nonemergency provider was assessed during the past 12 months (1 time, 2 times, 3 times, 4 times, 5-9 times, 10 or more times).

### Statistical Analyses

**Figure 1:**
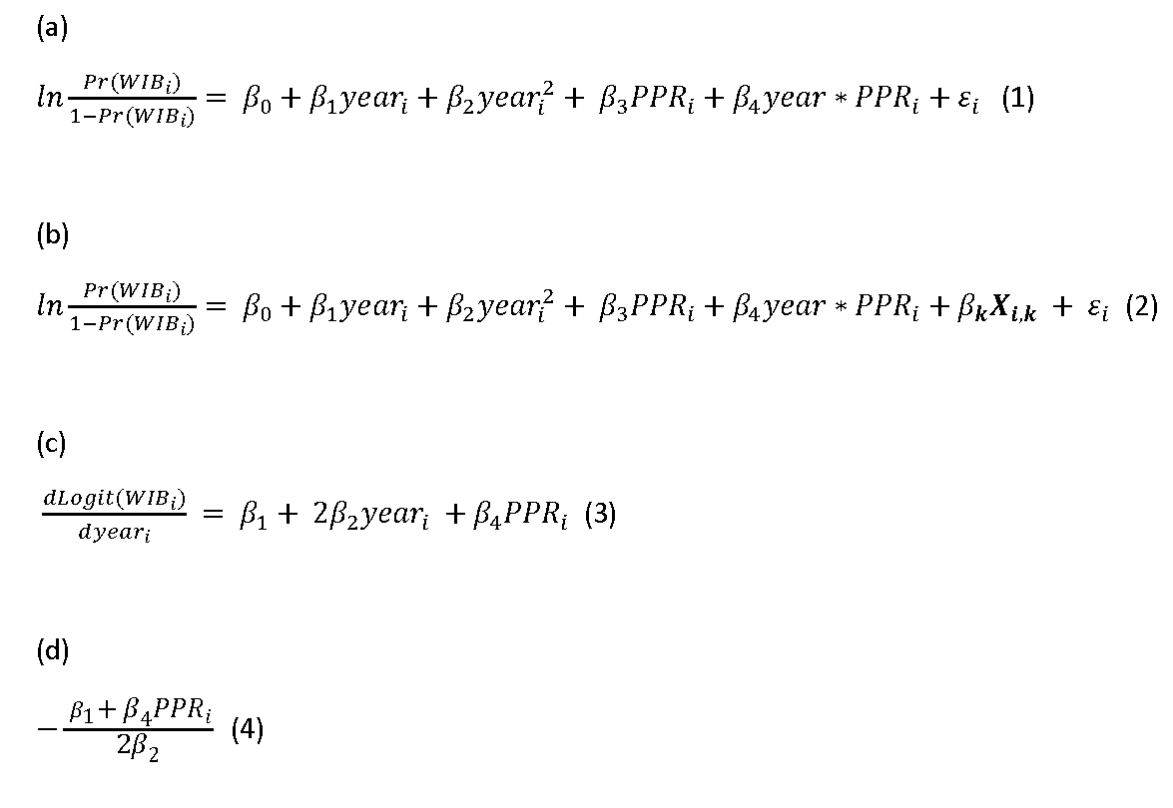
Equations for logistic regression models, slope, and change of point for time trends.

The study’s analytic goal is to examine the associations of our independent variables of interest (including PPR and its interaction term with time) with WIB across 5 time points. To achieve this goal, we utilized 2 logistic regression models structured as follows:

Model 1 is shown in equation 1 of Figure 1, where year is a continuous variable for time points and PPR represents PPR for respondent *i*. The quadratic term year _i_^2^ represents the curvilinear effect of year on logit (WIB). Model 2 is shown in equation 2 of Figure 1, where ***X*** is a vector of k control variables include gender, education, census division, urbanity, age group, occupation, whether born in the United States, general health, provider maintains electronic medical record, depression, trust in doctors, ever had cancer, and frequency to visit providers. The error term *ε_i_* is assumed to be independent of all independent variables and had a mean of zero.

The slope of time trend for logit of WIB is defined as the change of log-odds or logit per year and operationalized as shown in equation 3 of Figure 1, where PPR is treated as a constant. A change of point for the time trend is defined as the time when the slope is 0 and calculated as shown in equation 4 of Figure 1.

Odds ratio (OR) is defined as the exponential of the coefficient *β*(*e^β^*) Multicollinearity was inspected by calculating variance inflation factors (VIFs) for all variables. Findings resulted in VIFs lower than 5 among all independent and covariate variables, suggesting that multicollinearity is not at a concerning level [18]. The area under the receiver operating characteristics curve (C-statistics) was used to assess model performance and suggest sufficient model fit.

Chi-square tests were conducted to examine bivariate associations between outcome and demographic measures. All analyses were conducted using SAS version 9.4 (SAS Institute Inc). All descriptive analyses and logistic regression models were weighted to reflect a nationally representative estimate using the Statistical Analysis System procedures *Proc Surveyfreq* and *Proc Surveylogistic*, respectively. A 2-tailed *P*<.05 was considered statistically significant in this study.

## Results

Table 1 shows sample characteristics among study participants, which are stratified by their WIB status. Weighted proportions were calculated through the Jackknife estimation method to generalize findings to the population. Between 2011 and 2014, the proportioned differences between respondents with and without WIB increased from 2.29% to 4.61% and then dropped to −5.38% in 2018, indicating a potential nonlinear time trend of WIB. There are no significant differences between WIB and none-WIB groups when examining the gender and education sample distributions. Compared with respondents who did not withhold information from their providers, respondents who had WIB were more likely to be aged 25 to 44 years (difference: 13.82%) and employed (difference: 10.79%).

**Figure 2:**
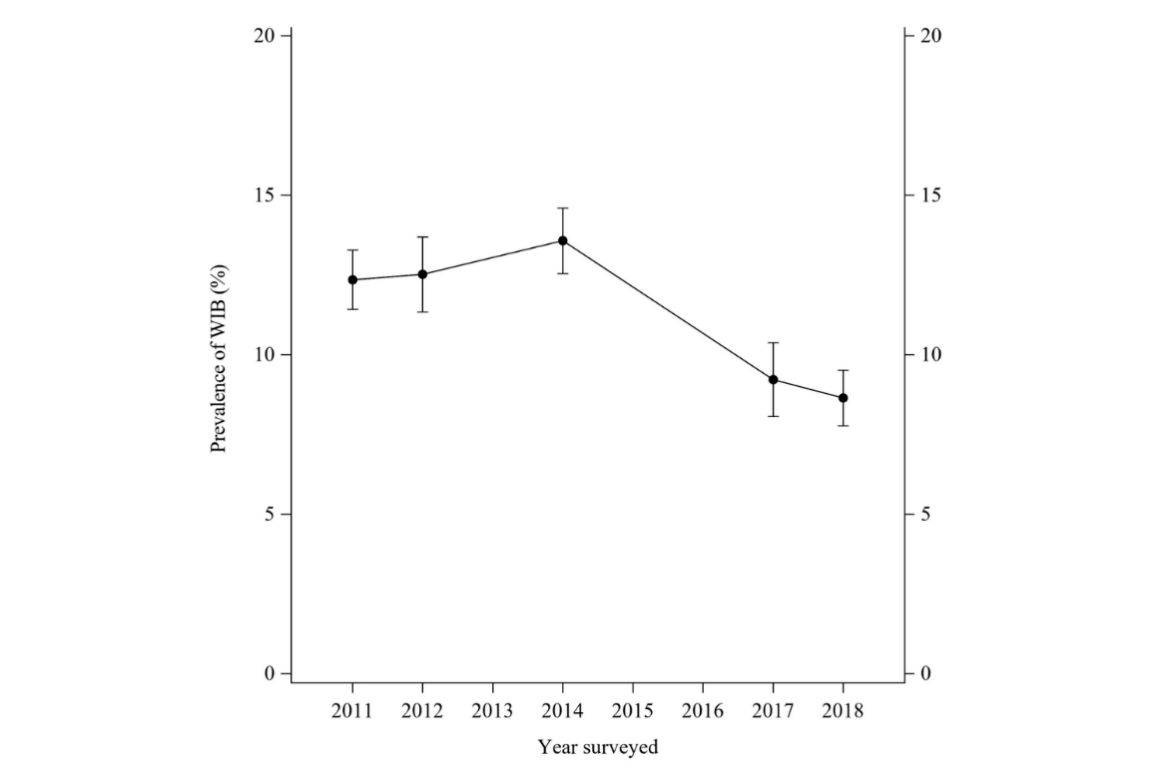
Unadjusted prevalence trend of withholding information behavior between 2011 and 2018 based on the Health Information National Trends Survey.

**Figure 3:**
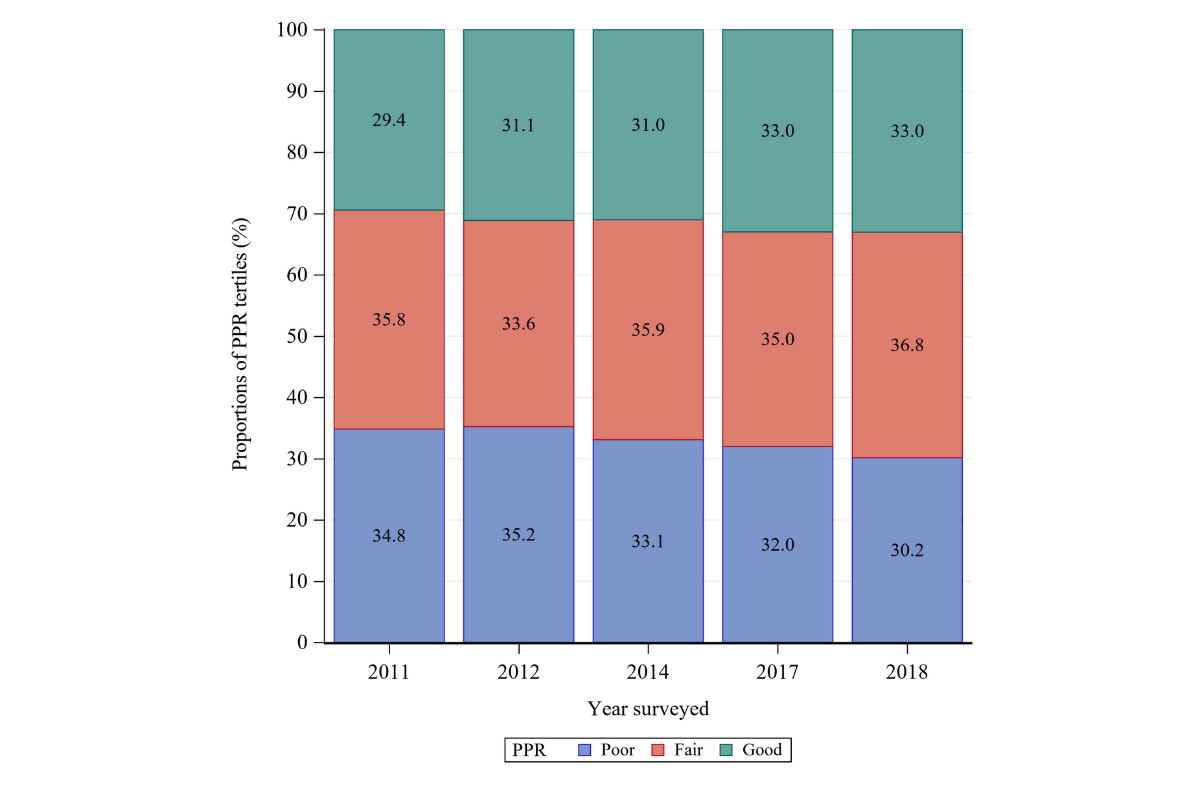
Unadjusted proportions of patient-physician relationship tertiles based on the Health Information National Trends Survey between 2011 and 2018. PPR: patient-physician relationship.

**Figure 4:**
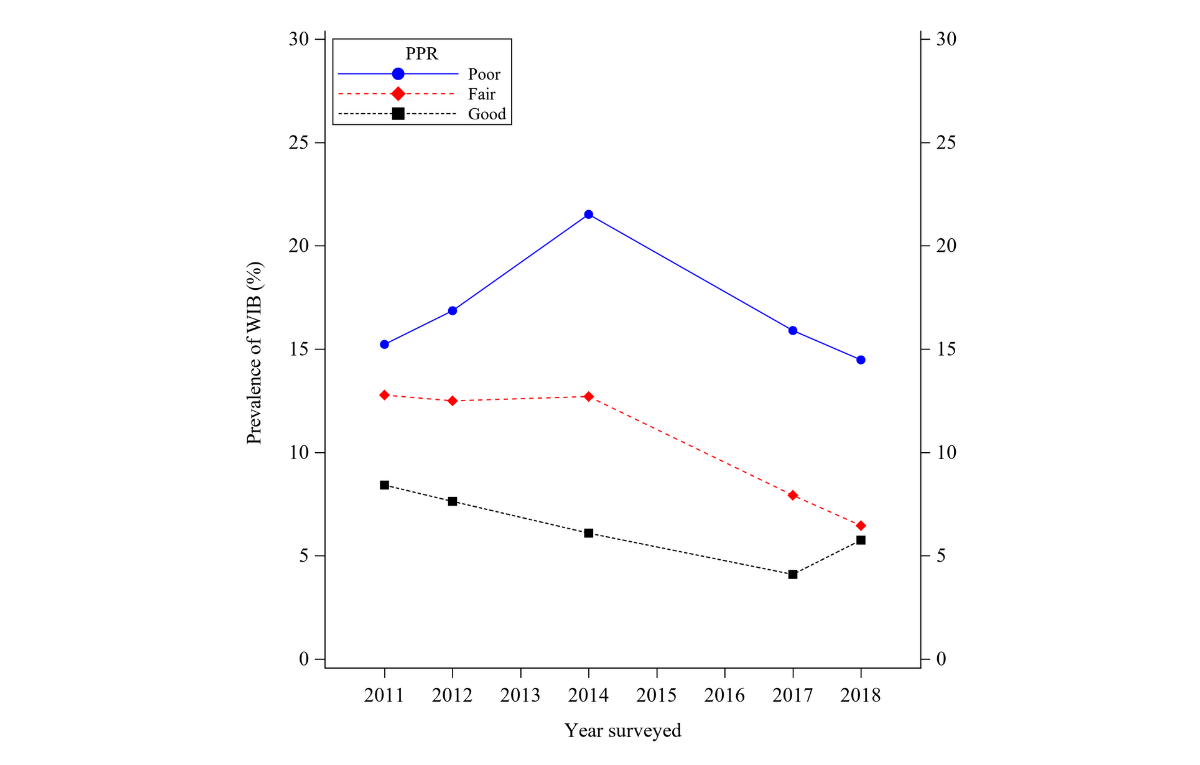
Unadjusted prevalence trends of withholding information behavior grouped by levels of patient-physician relationship and based on responses from the Health Information National Trends Survey between 2011 and 2018. WIB: withholding information behavior.

The weighted prevalence of WIB in the United States was assessed from 2011 to 2018. As Figure 2 shows, the WIB prevalence has an increasing trend before 2014, which has the highest rate of 13.6% and then decreased after 2014 to 8.6% (Multimedia Appendix 1). When examining the proportions of different levels of PPR across 5 time points, results show that PPR improves after 2011 as shown in Figure 3 by the increasing proportions of respondents with “good” PPR (tertile 3) and decreasing proportions of respondents with “poor” PPR (tertile 1). To explore the moderation effects of PPR on time trends for WIB (Figure 4), we plotted the weighted prevalence of WIB across time points by 3 categorized PPR levels. Results suggest that PPR affects the curvilinear time trend of WIB in 2 ways: on the one hand, the worse PPR is associated with an escalated prevalence of WIB in the year 2011; on the other hand, worse PPR also inverts the sign of the starting slope of curves, which is consistent with prior studies.

**Table 1:** Sample characteristics of the nationally representative sample across 5 iterations.

Variable		Withhold information, n (%): yes	Withhold information, n (%): no	Difference (%)	*P* value^a^
**Year**					.003
	2011	339 (21.5)	2357 (19.2)	2.29	
	2012	293 (21.7)	2018 (19.1)	2.62	
	2014	332 (23.2)	2039 (18.6)	4.61	
	2017	187 (17.3)	2002 (21.4)	−4.14	
	2018	199 (16.3)	2188 (21.7)	−5.38	
**Gender**					.14
	Male	500 (48.9)	4121 (45.8)	3.16	
	Female	850 (51.1)	6483 (54.2)	−3.16	
**Education**					.46
	Less than high school	79 (7.9)	691 (8.4)	−0.48	
	12 years or completed high school	182 (17.8)	1936 (19.5)	−1.67	
	Some college	451 (38.8)	3155 (35.5)	3.27	
	College graduate or higher	638 (35.5)	4822 (36.7)	−1.12	
**Age group**					<.001
	18-24	36 (4.8)	294 (10.8)	−5.97	
	25-44	462 (46.1)	2521 (32.3)	13.82	
	45-64	652 (41.0)	4506 (38.4)	2.6	
	65+	200 (8.1)	3283 (18.5)	−10.45	
**Occupation**					<.001
	Employed	838 (68.3)	5476 (57.5)	10.79	
	Unemployed	91 (7.0)	444 (5.5)	1.53	
	Retired	190 (7.8)	3163 (18.5)	−10.74	
	Disabled	121 (7.1)	670 (5.4)	1.76	
	Other	110 (9.7)	851 (13.1)	−3.34	

^a^*P* values are calculated from chi-square tests to show association between characteristics variables and WIB.

**Figure 5:**
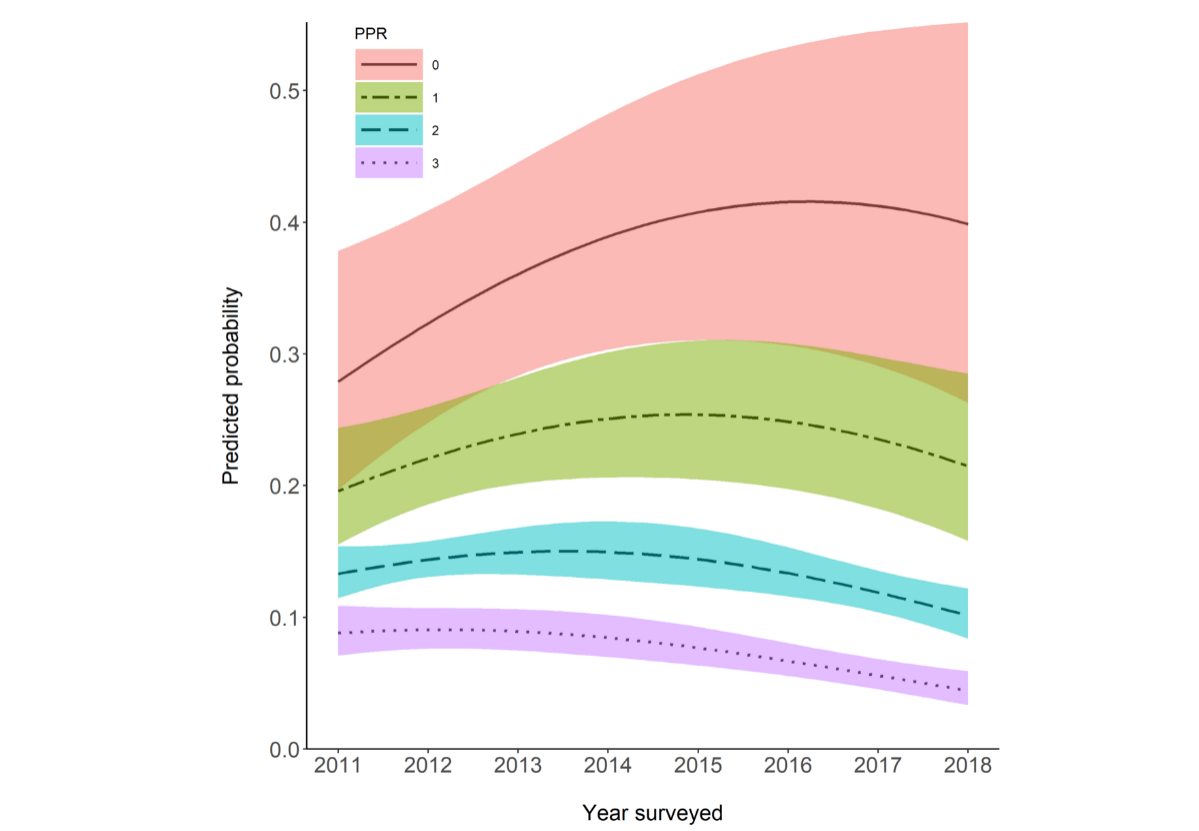
Predicted probability of withholding information behaviors at various patient-physician relationship values. PPR: patient-physician relationship.

For the multivariable logistic regression model, results displayed in Table 2 show the model coefficients and statistical significance for linear time trend, quadratic time trend, PPR, and interaction between linear time trend and PPR (model 1 as baseline model). A second logistic regression model (model 2) including covariates such as gender, age, and group education was used to examine whether coefficients were attenuated with the inclusion of confounding variables. The interaction term between quadratic time trend and PPR was not statistically significant and, therefore, removed from the model for simplicity of interpretation. Compared with the baseline model, model with covariates did not change the sign or strength of coefficient estimates, except PPR simple effect was about half of that in the baseline model (beta=−.286 in model 2 vs beta=−.462 in model 1). Accordingly, the odds for every 1 unit of PPR improvement as operationalized in our study is approximately a 24.9% decrease in withholding patterns (OR 0.751, 95% CI 0.598-0.943) in model 2 and 37% decrease (OR 0.63, 95% CI 0.518-0.766) in model 1 in the year 2011. For model 1, when holding PPR constant at 3 (the best PPR), the initial linear time trend slope was 0.052 (OR 1.053) and not significant (*P*=.48, not shown in the table). Due to the significant quadratic term (*P*=.04), the slope decreased by 0.044 per year, resulting in a decreasing trend after year 2012. However, when PPR was worse, for example, PPR=0, the linear time trend slope was significantly higher (beta=.233; SE=0.101; OR 1.263, 95% CI 1.030-1.547) and the change of point occurred later (about 5.3 years after 2011 when PPR=0). Figure 5 demonstrated visualization of the time trend for WIB probability, which was predicted by the logistic regression model 1, against the times surveyed at different PPR levels (0, 1, 2, and 3). PPR significantly modified time trend of WIB through increasing the initial slope in 2011 and, therefore, postponed the time for change of points.

**Table 2:** Coefficients of multiple logistic regression models with and without covariates.

Model and term		Coefficient estimates (beta)	SE	*P* value	Odds ratio and 95% CI
**Model 1: without covariates**					
	Intercept	−.950^a^	0.226	<.001	0.387 (0.246-0.609)^b^
	Year	.233	0.101	.03	1.263 (1.030-1.547)
	Year^2^	−.022	0.011	.04	0.978 (0.957-0.999)
	PPR^c^	−.462	0.097	<.001	0.630 (0.518-0.766)
	Year×PPR	−.060	0.025	.02	0.941 (0.896-0.989)
**Model 2: with covariates^d^**					
	Intercept	−1.763	0.58	.004	0.172 (0.053-0.551)
	Year	.253	0.104	.02	1.288 (1.044-1.588)
	Year^2^	−.024	0.011	.04	0.977 (0.955-0.999)
	PPR	−.286	0.113	.01	0.751 (0.598-0.943)
	Year×PPR	−.068	0.026	.01	0.934 (0.887-0.984)

^a^The estimate for the intercept is the baseline log-odds when year is in 2011 and PPR is 0.

^b^The odds ratio and 95% CI for intercept are the baseline odds and 95% CI when the year is in 2011 and patient-physician relationship is 0.

^c^PPR: patient-physician relationship.

^d^Covariates include gender, education, race, urbanity, age group, occupation, census division, born in the United States, general health status, provider maintains electronic medical record, depression, trust doctors, ever had cancer, and frequency to visit providers.

## Discussion

### Principal Findings

Our results suggest complex relationships between PPR and time trends of patients’ WIB in the United States. To the best of our knowledge, this study is the first to report the trend of WIB with close to a decade worth of responses (2011-2018). A previous report indicated that the proportion of individuals who withheld information from their providers were slightly decreased from 2012 to 2014, but the change was not statistically significant [10]. Our results revealed that the self-reported prevalence of withholding information first increased during 2011 to 2014 and then dropped to the lowest level in 2018. Although this study is not powered or designed to identify causal factors for the temporary increasing WIB between 2012 and 2014, one has considered whether the timing between this increase with the rise of health care data breaches between 2012 and 2015 [9] is beyond coincidence. Our data showed that PPR has improved steadily since 2011. As indicated by our logistic models, PPR is negatively associated with patients’ WIB, which supports our hypothesis that improved PPR lowers odds of patients exhibiting WIB patterns. Therefore, improved PPR may have led to the decreasing trend of WIB after 2014. However, we cannot rule out other unmeasured factors that contribute to the decreasing trend of WIB. Moreover, findings from this study highlight that the curvilinear time trend in WIB was moderated significantly by PPR, where our third focus was shown by the significant interaction term between year and PPR. In the population who reported the best PPR (PPR=3), the WIB prevalence starts to drop continuously as early as in 2012. When PPR was worse, the time for this change of point to occur was postponed to later years. Thus, improving PPR appears to have positive effects on reducing patients’ WIB both within and across time points surveyed.

It is not surprising that PPR is associated with patients’ WIBs. In Abdelhamid et al’s study [7], the PPR was found to be positively associated with the intention to share PHI electronically, which is consistent with our findings that better relationships lead to lower rates of WIB. In a qualitative study from a small sample of Malaysian, doctor’s interpersonal and communication skills were reported by all participants to affect their decisions to disclose medicine use information [14]. Another qualitative study among a Latino group found that low-quality relationships diminished participants’ willingness to disclose their health information [13]. In addition, existing findings suggest that (1) patients’ satisfaction with involvement in health care decisions and (2) perception of their doctors’ interest in their general health status also positively influence their decisions to share their eHealth findings [19]. Our study is consistent with these findings on the effects of PPR on patients’ disclosure behaviors and extends them to a nationally representative sample across multiple years. Due to the lack of literature in time trends analysis of WIB, our results for the first time reported the moderation effects of PPR on time trends, providing valuable information and insights for policy makers.

Although other factors such as demographics, socioeconomic status, and trust in doctors were not the focus of our study, we observed interesting findings that might be of interest to researchers in this area of study (Multimedia Appendix 2). For example, depression status is significantly associated with WIB in our study, which is consistent with previous observational research [5,20]. Other studies also reported that more depressive symptoms lead to decreased odds of patients disclosing medical information with their doctors [21,22]. Employment status as a dichotomous measure (ie, unemployed vs employed) was not found to be associated with WIB in previous research [5,20]. However, when including more subtypes of employment status, findings suggest that retired individuals were less likely to have WIB compared with individuals reporting as currently employed. This relationship among retired Americans is consistent with our results that older adults have lower odds of WIB than that of middle-aged population. A previous study showed that the top reasons for patients’ failures to disclose information are related to trust in clinicians and stigmatization of health behavior [4]. The literature recognizes trust as a factor for improving patients willingness to participate in research and share information [23-27]. The association between WIB and factors such as trust in doctors, PPR, and depression status revealed that these factors may be critical contributors to WIB. Therefore, efforts are warranted for improving relationships between patients and providers and reducing self-stigmatization of patients who have mental diseases to reduce the likelihood of patients’ WIB. We did not find any statistically significant interaction effects between PPR and these factors on WIB or the interaction between year and these factors. If consistent in future studies, these findings suggest that providers working with patients at risk for mental health conditions may need to be extra mindful of communicative interactions to reduce odds of WIB patterns.

### Limitations and Strength

Similar to other observational studies, this study also has limitations because of the nature of sampling design. First, our analyses were cross-sectional in nature, though we included multiple years of surveys in the trend analysis. The association between predictors and WIBs was not supported by causal inference; therefore, findings should be interpreted carefully and considered as evidence supporting the allocation of resources to examine WIB using a randomized controlled trial research design. Second, HINTS were based on self-reported responses, which are subject to social desirability bias and measurement errors [28]. However, this limitation may be remedied by the stratified random sampling and weighting techniques common in nationally representative complex sampling surveys. Third, our study may be limited by not including various covariates unavailable in the HINTS study. Conversely, a strength of this study is that findings were produced from multiple years of HINTS data to track WIB at a national level. This advantage allowed us to assess moderation effects of PPR on WIB trends, which cannot be detected with limited years of HINTS data [5]. Another strength of our study is that we examined the curvilinear time effects, which allow for a more accurate presentation of WIB over multiple years. Previous research appears overly reliant on statistical methods that assume a linear relationship between continuous predictors and logit of outcome in a logistic regression, which obscures the curvilinear patterns of predictors and renders their models less representative for actual data [29]. In addition, our analyses on “change of points” integrates measures of interest using polynomial terms, which is suggested to unveil useful information for policy makers and practitioners [30].

### Conclusions

We found that there is a curvilinear time trend for the prevalence of patients’ WIB. In addition, we found that the PPR is significantly associated with whether patients withhold information from their providers. Moreover, our analyses supported that PPRs moderate the time trends of withholding behavior, and the low quality of relationships between patients and providers postpones the change of point for the decreasing trend. As previously mentioned, the findings from this study in and of itself are not sufficient to motivate changes in national policy. Nevertheless, we believe our findings are interesting enough to warrant further investigation, and if reproducible, the study reinforces the support for interventions bolstering the PPR. Therefore, if findings remain similar in future studies, events and other factors that lower chances of patients fully divulging critical health information can be reduced through improved PPRs. To advance research in this area of study, we believe it to be prudent for future research to replicate this study design with WIB and PPR responses among patients collected on a more granular timescale (eg, “day” and “week”), with inclusion of specific medical breaches to examine moderating effects of medical breaches. The withholding of one’s health behavior during medical visits has serious implications on population health. As such, ways to reduce this behavior are of great importance to society.

## Appendix

Multimedia Appendix 1 Weighted prevalence and 95% confidence intervals of withholding information behavior across 5 survey years.

Multimedia Appendix 2 Odds ratio and 95% confidence intervals of covariates in a multiple logistic regression models (model 2).

## Abbreviations

eHealth: electronic health
HINTS: Health Information National Trends Survey
OR: odds ratio
PHI: protected health information
PPR: patient-physician relationship
WIB: withholding information behavior
VIF: variance inflation factor
