# Supplementary material for: Effect of Patient-Physician Relationship on Withholding Information Behavior: Analysis of Health Information National Trends Survey (2011-2018) Data
Source: J Med Internet Res. 2020 Jan 29;22(1):e16713. doi: 10.2196/16713 (PMC7016621; doi:10.2196/16713)
Supplement: Multimedia Appendix 1 [file jmir_v22i1e16713_app1.docx]

| Multimedia Appendix 1: Weighted prevalence and 95% confidence intervals of WIB across five survey years | | | | | |
| --- | --- | --- | --- | --- | --- |
| Year surveyed | Frequency | Weighted prevalence (%) | Lower 95% confidence limit (%) | Upper 95% confidence limit (%) |  |
| 2011 | 339 | 12.3 | 10.5 | 14.2 |  |
| 2012 | 293 | 12.5 | 10.2 | 14.9 |  |
| 2014 | 332 | 13.6 | 11.5 | 15.6 |  |
| 2017 | 187 | 9.2 | 6.9 | 11.5 |  |
| 2018 | 199 | 8.6 | 6.9 | 10.4 |  |
