# Supplementary material for: Effect of Patient-Physician Relationship on Withholding Information Behavior: Analysis of Health Information National Trends Survey (2011-2018) Data
Source: J Med Internet Res. 2020 Jan 29;22(1):e16713. doi: 10.2196/16713 (PMC7016621; doi:10.2196/16713)
Supplement: Multimedia Appendix 2 [file jmir_v22i1e16713_app2.docx]

| Multimedia Appendix 2: Odds ratio and 95% confidence intervals of covariates in a multiple logistic regression models (model 2) | | | |
| --- | --- | --- | --- |
| Variable |  | ORs odds ratio(95% CI) |  |
|  | | |  |
| Gender | Male | Ref. *P =.15* |  |
|  | Female | 0.87 (0.73,1.05) |  |
|  | | |  |
| Education | Less than high school | Ref. *P =.07* |  |
|  | 12 years or completed high school | 1.14 (0.73,1.78) |  |
|  | Some college | 1.37 (0.91,2.07) |  |
|  | College graduate or higher | 1.08 (0.69,1.71) |  |
|  | | |  |
| Census division | New England | 0.95 (0.53,1.69) |  |
|  | Middle Atlantic | 1.33 (0.91,1.95) |  |
|  | East North Central | Ref. *P =.39* |  |
|  | West North Central | 1.33 (0.74,2.41) |  |
|  | South Atlantic | 1.02 (0.74,1.42) |  |
|  | East South Central | 0.90 (0.56,1.43) |  |
|  | West South Central | 1.04 (0.69,1.58) |  |
|  | Mountain | 1.37 (0.84,2.21) |  |
|  | Pacific | 1.31 (0.90,1.91) |  |
|  | | |  |
| Race | Hispanic | Ref. *P =.05* |  |
|  | Non-Hispanic White | 0.74 (0.51,1.08) |  |
|  | Non-Hispanic Black | 1.02 (0.67,1.57) |  |
|  | Non-Hispanic Other | 0.99 (0.52,1.91) |  |
|  | Non-Hispanic Asian | 1.26 (0.77,2.08) |  |
|  | | |  |
| Urban/Rural | Urban | Ref. *P =.80* |  |
|  | Rural | 1.04 (0.74,1.47) |  |
|  | | |  |
| Age group | 18-24 | Ref. *P < .001* |  |
|  | 25-44 | 2.87 (1.63,5.04) |  |
|  | 45-64 | 2.54 (1.50,4.31) |  |
|  | 65+ | 1.65 (0.89,3.07) |  |
|  | | |  |
| Occupation | Employed | Ref. *P =.008* |  |
|  | Unemployed | 0.84 (0.50,1.39) |  |
|  | Retired | 0.54 (0.37,0.79) |  |
|  | Disabled | 0.90 (0.56,1.46) |  |
|  | Other | 0.74 (0.54,1.02) |  |
|  | | |  |
| Born in US | Yes | Ref. *P =.36* |  |
|  | No | 1.15 (0.85,1.54) |  |
|  | | |  |
| General health | Excellent | Ref. *P =.93* |  |
|  | Very good | 0.94 (0.62,1.43) |  |
|  | Good | 0.87 (0.54,1.39) |  |
|  | Fair | 1.00 (0.62,1.62) |  |
|  | Poor | 0.84 (0.42,1.67) |  |
|  | | |  |
| Provider maintain EMR | Yes | Ref. *P =.58* |  |
|  | No | 0.85 (0.56,1.29) |  |
|  | Don't know | 0.87 (0.44,1.74) |  |
|  | | |  |
| Depression | Normal | Ref. *P < .001* |  |
|  | Mild | 1.28 (0.97,1.67) |  |
|  | Moderate | 2.09 (1.52,2.88) |  |
|  | Severe | 1.46 (0.92,2.32) |  |
|  | | |  |
| Trust doctors | A lot | Ref. *P =.003* |  |
|  | Some | 1.43 (1.08,1.89) |  |
|  | A little | 1.66 (1.01,2.74) |  |
|  | Not at all | 2.22 (0.88,5.63) |  |
|  | | |  |
| Ever had cancer | Yes | Ref. *P =.80* |  |
|  | No | 0.97 (0.74,1.27) |  |
|  | | |  |
| Frequency go provider | 1 time | Ref. *P =.52* |  |
|  | 2 times | 1.10 (0.79,1.52) |  |
|  | 3 times | 0.91 (0.59,1.41) |  |
|  | 4 times | 1.08 (0.76,1.53) |  |
|  | 5-9 times | 1.13 (0.75,1.71) |  |
|  | 10 or more times | 1.35 (0.87,2.09) |  |
